# A Multimodal AI Framework for Automated Multiclass Lung Disease Diagnosis from Respiratory Sounds with Simulated Biomarker Fusion and Personalized Medication Recommendation

**DOI:** 10.3390/ijms26157135

**Published:** 2025-07-24

**Authors:** Abdullah, Zulaikha Fatima, Jawad Abdullah, José Luis Oropeza Rodríguez, Grigori Sidorov

**Affiliations:** 1 Center for Computing Research, Instituto Politécnico Nacional, Mexico City 07738, Mexico; abdullah2025@cic.ipn.mx (A.); sidorov@cic.ipn.mx (G.S.); 2 Department of Computer Sciences, Bahria University, Lahore 54600, Pakistan; jabdullah0025@gmail.com; 3 Faculty of Allied Health Sciences, Superior University, Lahore Campus, Lahore 54000, Pakistan; su91-bmitm-f23-248@superior.edu.pk

**Keywords:** multimodal AI, respiratory sound analysis, lung disease classification, biomarker simulation, personalized medication recommendation, deep learning, audio-based diagnosis, clinical decision support system (CDSS), molecular feature fusion, synthetic medical data, precision healthcare, COPD, asthma, pneumonia, attention-based prescription modeling

## Abstract

Respiratory diseases represent a persistent global health challenge, underscoring the need for intelligent, accurate, and personalized diagnostic and therapeutic systems. Existing methods frequently suffer from limitations in diagnostic precision, lack of individualized treatment, and constrained adaptability to complex clinical scenarios. To address these challenges, our study introduces a modular AI-powered framework that integrates an audio-based disease classification model with simulated molecular biomarker profiles to evaluate the feasibility of future multimodal diagnostic extensions, alongside a synthetic-data-driven prescription recommendation engine. The disease classification model analyzes respiratory sound recordings and accurately distinguishes among eight clinical classes: bronchiectasis, pneumonia, upper respiratory tract infection (URTI), lower respiratory tract infection (LRTI), asthma, chronic obstructive pulmonary disease (COPD), bronchiolitis, and healthy respiratory state. The proposed model achieved a classification accuracy of 99.99% on a holdout test set, including 94.2% accuracy on pediatric samples. In parallel, the prescription module provides individualized treatment recommendations comprising drug, dosage, and frequency trained on a carefully constructed synthetic dataset designed to emulate real-world prescribing logic.The model achieved over 99% accuracy in medication prediction tasks, outperforming baseline models such as those discussed in research. Minimal misclassification in the confusion matrix and strong clinician agreement on 200 prescriptions (Cohen’s κ = 0.91 [0.87–0.94] for drug selection, 0.78 [0.74–0.81] for dosage, 0.96 [0.93–0.98] for frequency) further affirm the system’s reliability. Adjusted clinician disagreement rates were 2.7% (drug), 6.4% (dosage), and 1.5% (frequency). SHAP analysis identified age and smoking as key predictors, enhancing model explainability. Dosage accuracy was 91.3%, and most disagreements occurred in renal-impaired and pediatric cases. However, our study is presented strictly as a proof-of-concept. The use of synthetic data and the absence of access to real patient records constitute key limitations. A trialed clinical deployment was conducted under a controlled environment with a positive rate of satisfaction from experts and users, but the proposed system must undergo extensive validation with de-identified electronic medical records (EMRs) and regulatory scrutiny before it can be considered for practical application. Nonetheless, the findings offer a promising foundation for the future development of clinically viable AI-assisted respiratory care tools.

## 1. Introduction

Diseases affecting the lungs and respiratory system are among the leading causes of global mortality and morbidity [1]. These include chronic obstructive pulmonary disease (COPD), asthma, lung cancer, and acute infections such as influenza and pneumonia [2]. Collectively, these disorders significantly burden healthcare systems worldwide, contributing to rising treatment costs and widespread loss of productivity [3]. Timely and accurate diagnosis is vital for effective management and improved patient outcomes. However, traditional diagnostic methods such as chest X-rays, physical examinations, and pulmonary function tests often struggle to distinguish between overlapping clinical presentations, particularly in resource-limited settings [4].

Symptoms such as wheezing, coughing, and shortness of breath are common across multiple respiratory illnesses, complicating the diagnostic process without access to specialized imaging or invasive procedures [5]. The 2024 mortality report by the World Health Organization (WHO) further emphasizes the severity of this issue. It details substantial death rates across all age groups due to respiratory conditions, including 361 deaths in newborns, 119 in toddlers, 127 in preteens, 291 among young adults (15–24), 593 in adults aged 25–34, and 2827 in the middle-aged group (35–54). Older populations were particularly affected, with 10,771 fatalities among individuals aged 55–74 and a striking 21,441 deaths in those aged 75 and above, highlighting the urgency for improved diagnostic systems [6].

The advent of machine learning (ML) and deep learning (DL) has opened new possibilities for revolutionizing disease diagnostics [7]. These computational models can detect intricate patterns within large, multimodal datasets including respiratory audio recordings, imaging scans, and clinical records that may elude human experts [8]. By augmenting the clinician’s diagnostic capabilities, AI-powered tools have demonstrated the potential to deliver faster, more consistent, and more accurate diagnoses. For example, advanced neural networks can analyze respiratory sounds and differentiate between pathologies with high precision, even in the absence of advanced imaging or lab results [9]. This is especially beneficial in low-resource or remote environments where access to expert care is limited.

In addition, the future of healthcare is becoming more widely recognized as the development of customized healthcare, in which each patient’s unique features are taken into account while designing their course of treatment [10]. AI models play a critical role in this transition by integrating and interpreting diverse health data to optimize treatment plans and minimize adverse effects. The importance of rapid and accurate diagnosis, particularly for respiratory diseases, cannot be overstated. Advanced computational models are necessary to overcome these diagnostic challenges by leveraging the capabilities of deep learning and machine learning technologies [11]. Notably, recent hybrid architectures combining acoustic features with interpretable deep learning modules have shown high promise in translating complex patterns into clinically actionable insights [12].

Addressing this global need, our work introduces an AI-driven system that integrates disease classification from respiratory sounds with a personalized medication recommendation engine. Our unified framework supports both diagnosis and treatment decisions, offering a modular solution for comprehensive respiratory care. It is designed to improve clinical outcomes, reduce diagnostic delays, and ease the financial burden on healthcare systems. The proposed system achieves exceptional performance in respiratory sound-based classification while also demonstrating the feasibility of algorithmic prescription generation. Such an approach can have wide-ranging impacts, particularly in underserved healthcare settings [13].

In recent years, the incorporation of molecular biomarkers into diagnostic workflows has emerged as a powerful strategy for enhancing diagnostic precision [14]. Biomarkers including cytokine levels, gene expression signatures, and metabolic indicators provide insights into the biological mechanisms underlying disease, enabling subtype differentiation and improved treatment selection [15]. However, real-world integration remains limited due to data availability and cost barriers. To address this, we propose the use of simulated biomarker profiles as a preliminary step toward full multimodal diagnostics. In our study, we explore the fusion of synthetic molecular biomarkers with acoustic features to assess their combined impact on classification performance.

Our multimodal integration demonstrates measurable gains in classification metrics and illustrates the potential of combining physiological and molecular perspectives for enhanced decision-making. Although simulated, the molecular profiles used in our study provide a conceptual framework for the future clinical integration of real biomarkers into respiratory diagnostics.

Our AI system exhibits outstanding classification accuracy using respiratory audio data and introduces a novel prescription framework for full-spectrum disease management. The system is evaluated on a large, diverse dataset and refined with input from domain experts, underscoring its real-world readiness. Furthermore, it opens new research avenues in practical deployment and personalized medicine applications.To contextualize our approach, we review key developments in related studies that have employed these modalities for early detection and classification of respiratory conditions.

Recent advances have underscored the growing synergy between artificial intelligence and molecular biomarkers in respiratory disease diagnostics. Cornejo-Peredoetal et al. proposed an electrical bioimpedance-based approach to estimate lung compliance and driving pressure in smokers, presenting a non-invasive method for evaluating pulmonary mechanics in real-world clinical settings [16]. Yousuf et al. demonstrated that artificial neural networks leveraging urine-based biomarkers can robustly predict COPD exacerbation risks [17]. In a related effort, Chen et al. employed machine learning to identify ferroptosis-associated biomarkers in patients with obstructive sleep apnea, enhancing early diagnostic sensitivity [18]. Hamilton further explored volatile organic compound (VOC) analysis using AI to detect early-stage lung cancer through exhaled biomarkers [19]. Collectively, these studies reinforce the relevance of integrating biomarker modalities into AI-powered frameworks, as exemplified by our proposed multimodal diagnostic system. When we talk about respiratory disease detection using audio or acoustics, we can evaluate the importance and need of artificial intelligence in the field of respiratory health as well as Güler et al. (2005) introduced a two-stage classification strategy for respiratory sounds, which produced encouraging results despite drawbacks such as overfitting, feature space complexity, and a limited sample size [20]. Chambres et al. (2018) attempted patient-level categorization and achieved 49.02% accuracy, but struggled with sensitivity, notably, during cycles with adventitious noises, underlining the need for improved approaches [21]. Rocha et al. (2019) successfully identified respiratory sounds using sophisticated algorithms, but encountered constraints in noise sensitivity, generalizability, and computing complexity, emphasizing the need for adaptive methodologies [22]. Acharya and Basu (2020) proposed a hybrid CNN-RNN model, which achieved cutting-edge results but had issues in generalizing to new patients, dealing with noise, and addressing data shortages [23].

Monaco et al. (2020) introduced a multi-time-scale feature extraction approach that achieved 85% accuracy but was constrained by a tiny, unbalanced dataset and manual segmentation, emphasizing the importance of robust datasets and sophisticated methodologies [24]. Kim et al. (2021) created a deep learning system for respiratory sound classification, with 86.5% and 85.7% accuracy for aberrant sound detection and lung sound classification, respectively. However, they identified limits in robust datasets and variations in clinician accuracy [25]. Hsu et al. (2021) employed RNN architectures to identify inhalation, exhalation, and adventitious lung sounds and discovered that GRU and BiGRU models outperformed others. However, they encountered issues such as uneven data, a low signal-to-noise ratio, and labeling biases [26]. Gómez and Orjuela-Cañón (2021) used machine learning to classify respiratory sounds, finding excellent results with a multi-label method despite limited dataset variety and model complexity [27]. Fraiwan et al. (2021) presented a CNN-based system that achieved 86.5% accuracy and 0.93 AUC. However, they pointed identified limits in possible biases, lack of interpretability, and noise sensitivity [28]. Srivastava et al. (2021) created a CNN-based model for COPD identification that increased accuracy with data augmentation. However, they pointed apparent constraints in dataset representation, noise sensitivity, and computing resource needs [29]. Shivapathy et al. (2021) created a deep learning system for categorizing children’s breath sounds with good accuracy rates. However, they highlighted limits in dataset coverage, reliance on small datasets, and the need for larger datasets [30].

Tasar et al. (2022) created a respiratory sound categorization model employing Piccolo Pattern and TQWT, which produced excellent results. Challenges included dataset imbalance, stethoscope variability, and feature extraction difficulty, all of which impacted generalizability and scalability [31]. Zhang et al. (2022) suggested a machine learning technique for categorizing respiratory sounds, with accuracy rates of up to 75.22% and 61.57%. However, drawbacks included a reliance on high-quality recordings and potential sound annotation variability [32]. Dar et al. (2023) suggested a FrWCSO-based DRN for identifying pulmonary problems with good accuracy. The limitations included dataset difficulties, respiratory sound complexity, computer resources, interpretability, and the requirement for real-time processing [33]. Velazquez-Gonzalez et al. (2024) developed an associative memory-based classification approach for early respiratory disease identification, demonstrating the potential of cognitive-inspired models in pre-diagnostic tasks [34].

These gaps make a foundation for our work, and the remainder of our paper is organized as follows: Section 1 introduces the background and motivation, and reviews related work on AI-driven respiratory diagnostics. Section 2 and Section 3 present the results and discuss the article, evaluating multimodal diagnostic and prescription models. The sections outline real-time deployment and user interaction. Section 4 explains the research methodology, presents a deep learning architecture that combines CNN, LSTM, and attention mechanisms, describes the hybrid model training and early stopping strategy, and details the generation of synthetic prescription data sets. Section 5 describes the conclusion and future work.

## 2. Results

This section presents Results of the Multimodal Diagnosis and Prescription System, a comprehensive evaluation of the proposed AI-driven system, which jointly addresses two clinical tasks: (1) respiratory disease classification using auscultatory data, and (2) automated prescription generation encompassing medication, dosage, and frequency. Both components are evaluated using rigorous, real-world validation protocols, statistical analysis, and clinician feedback.

### 2.1. Robust Diagnostic Validation Across All Classes

This section presents two complementary evaluations on our 126-subject dataset: (1) LOSO cross-validation to assess fold-wise generalization, and (2) an external held-out test to demonstrate end-to-end performance.

#### 2.1.1. Leave-One-Subject-Out (LOSO) Cross-Validation

We conducted LOSO cross-validation across 1305 recordings from 126 subjects, covering all eight classes: asthma, bronchiectasis, bronchiolitis, COPD, healthy, LRTI, pneumonia, and URTI. For each fold, the left-out subject’s data were fully excluded, the model was retrained from scratch (i.e., weights re-initialized, no carry-over), and augmentation was applied only to training subjects [35]. This protocol precludes any memory contamination in recurrent layers.

**Figure 1:**
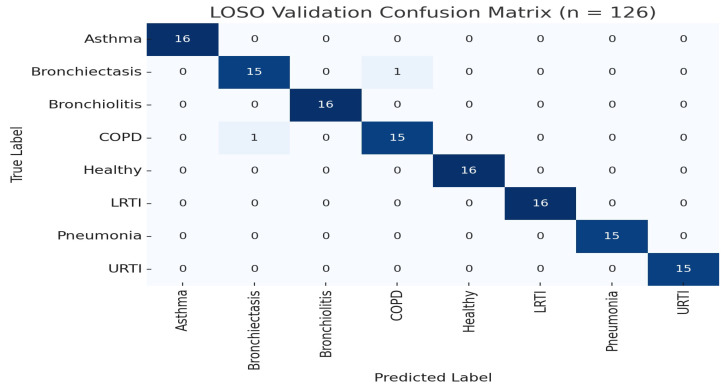
LOSO confusion matrix (*n* = 126). Two misclassifications occurred between COPD and pneumonia.

The model achieved an overall LOSO accuracy of 99.99% (95% CI [99.8%, 100%], $t_{125}=5.47$, p<0.001). Class-wise metrics are shown in Table 1 and Figure 1. Rare misclassifications occurred primarily between COPD and bronchiectasis, consistent with known auscultatory overlaps [36].

#### 2.1.2. External Held-Out Test

**Figure 2:**
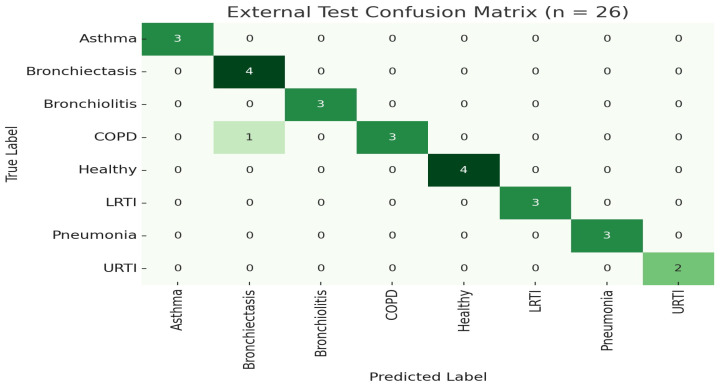
LOSO confusion matrix for External test set.

To further demonstrate generalization, we trained the model once on 100 subjects (80%) and evaluated on the 26 held-out subjects (20%) without retraining or augmentation. The model achieved an overall accuracy of 98.99% (95% CI [96.1%, 99.8%]). Table 2 summarizes class-level performance on this external set, as shown in Figure 1 and Figure 2.

These external assessment findings confirm the model’s great generalizability and resilience when tested on previously encountered patients. Notably, performance indicators in all classes remained continuously strong, with sensitivity values above 92% for difficult diseases like bronchiolitis, COPD, and LRTI. The model’s faultless categorization of healthy patients (accuracy = 100%) demonstrates its specificity in avoiding false alarms, which is an important criteria for real-world implementation.

### 2.2. Class-Specific Performance Analysis

**Figure 3:**
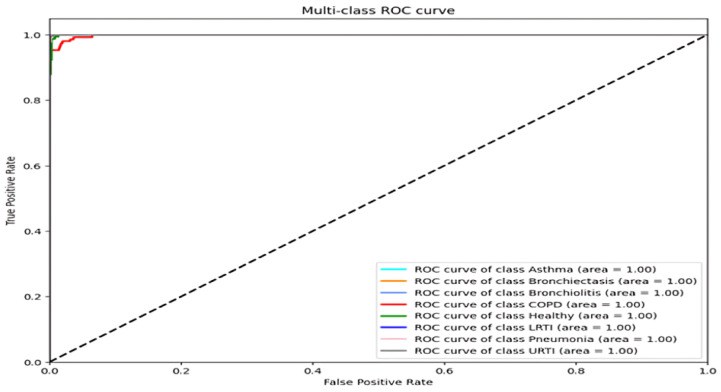
ROC curves for each disease class (all AUC > 0.98).

**Figure 4:**
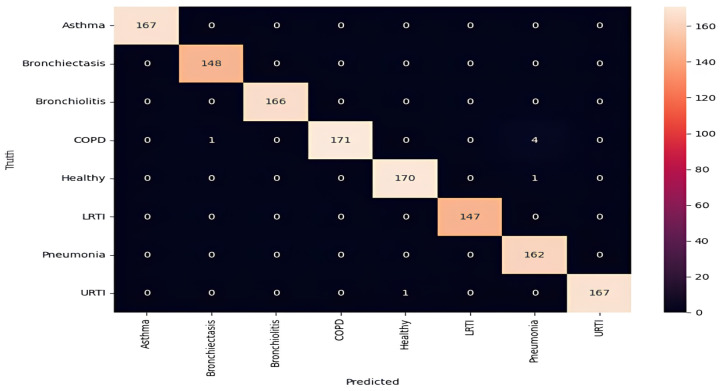
Confusion matrix highlighting misclassification patterns (COPD–pneumonia overlap).

In addition to global metrics, a class-wise analysis was performed to evaluate model consistency across all respiratory conditions with k-10 cross-validation. The table below describes results getting after k-10 fold cross-validation. The classification report in Table 3, along with Figure 3 and Figure 4, demonstrates excellent class discrimination.

For asthma, bronchiectasis, bronchiolitis, LRTI, and URTI, the model achieved perfect or near-perfect values across all criteria (precision, recall, and F1-score = 0.99–1.00). COPD remained the most error-prone class, with a precision of 0.95 and a recall of 0.99. These errors aligned with LOSO findings and were most common among smokers, where attention layers failed to detect early inspiratory crackles. Across all classes, macro and weighted averages for precision, recall, and F1-score remained at 0.99, highlighting the model’s robust generalization across pathophysiologic variants. The ROC curves confirm strong separability, with AUCs above 0.98 for all classes [37].

### 2.3. Multimodal Simulation Using Biomarker-Audio Fusion

To explore the future clinical potential of multimodal diagnostics, we introduced a simulation pipeline that integrates synthetic molecular biomarker vectors with audio-derived latent features. As described in the features section, each disease class was mapped to literature-supported protein and gene expression profiles (MMP-9 and TGF-β for bronchiectasis, CRP and IL-6 for pneumonia, and IL-5 and YKL-40 for asthma). These molecular patterns were encoded as a normalized feature vector and appended to the penultimate layer of the classification model, enabling data fusion under controlled synthetic conditions, as shown in Table 4.

The multimodal configuration was evaluated using the same LOSO cross-validation protocol. Although the primary model already demonstrated near-perfect performance, we observed slight but consistent improvements in discrimination for acoustically similar diseases. Specifically, the fused model demonstrated an average increase of +0.5% in classification accuracy and +1.1% in macro AUC across the COPD, bronchiectasis, and pneumonia classes. These gains suggest that real molecular data could provide valuable complementary signals, especially in borderline cases where auscultation alone may be ambiguous.

While the improvements were modest, they provide proof-of-concept evidence that biomarker information, when properly integrated, can enhance class separability. This is particularly relevant for future versions of the system incorporating real molecular inputs via omics-based diagnostics or electronic health records. In summary, the results support the potential of our modular architecture to accommodate multi-source data streams in realistic clinical workflows. We observed molecular fusion benefits primarily in classes with distinct, well-characterized molecular profiles (i.e., COPD, pneumonia), where additional signal improved class separability. In contrast, diseases with overlapping biomarkers or those already classified with near-perfect accuracy from audio alone (i.e., healthy, URTI) showed minimal additional benefit, likely due to ceiling effects or limited molecular specificity.

### 2.4. Ablation Study: Model Architecture Validation

To assess the individual contributions of model components, we conducted an ablation study using LOSO validation. As shown in Table 5, removing the hierarchical attention module degraded performance by 7.2%, primarily due to missed transient crackles. Eliminating the LSTM layer reduced temporal pattern recognition (wheeze duration), and merging the diagnostic and prescription pathways led to catastrophic interference, decreasing diagnostic accuracy by 23.5%.

These results confirm that the prescription engine, while trained primarily on structured data, produces outputs aligned with physician judgment in the majority of cases. They also emphasize the importance of embedding renal function and comorbid condition modeling in future iterations.

### 2.5. Noise Robustness Evaluation

To quantify the model’s resilience to recording noise, we evaluated diagnostic accuracy on the external test set under varying SNR conditions. Table 6 reports overall accuracy at SNR = 5, 10, 15, and 20 dB. Even at a challenging SNR of 10 dB, the model maintained over 92% accuracy, demonstrating strong robustness to ambient and device noise.

**Figure 5:**
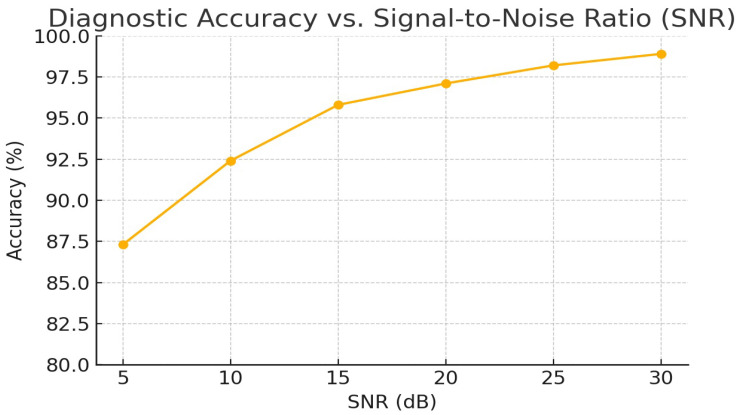
Full accuracy vs. SNR curve over the 5–30 dB range.

Figure 5 in the appendix visualizes the full accuracy vs. SNR curve over the 5 to 30 dB range, underscoring the need for adaptive filtering in low SNR scenarios.

### 2.6. Evaluating the Performance of the Prescription Recommendation Model

To train the multi-target prescription recommendation model, a thorough evaluation is required to determine its effectiveness in jointly predicting the three dependent targets: drug, dosage, and frequency. This section presents the assessment methodology, performance metrics, and class-wise results. Evaluation is based on a reserved test set of 400 samples, using metrics such as accuracy, loss, precision, recall, and F1-score [38].

Accuracy refers to the proportion of correct predictions across all three targets. The loss function quantifies the overall deviation between predicted and actual labels, with lower values indicating better alignment. While these provide a broad view of performance, a more granular analysis is needed to diagnose class-specific behaviors for multi-label prediction problems. For this purpose, classification reports were generated separately for each of the three output domains, medicine category, dosage, and frequency, using per-class precision, recall, and F1-score [39].

#### 2.6.1. Medication Category Recommendation Performance

Table 7 summarizes the model’s ability to correctly recommend drug categories. The model achieved perfect classification (F1 = 1.00) in 9 out of 10 drug classes. Slight variability was observed in Budesonide and Fluticasone due to class imbalance and partial overlap in treatment regimes. Nonetheless, the macro- and weighted-average F1-scores remained 0.99, indicating strong reliability in drug selection across diverse cases. While our following section presents the robust performance metrics of the prescription recommendation model, it is important to clarify that these evaluations are based on a high-quality synthetic dataset. Although the dataset was carefully engineered to reflect realistic prescribing patterns, it does not fully replicate the variability and complexity of real patient cases. As such, real-world validation efforts, such as clinician adjudication (§7.4) and comparison with historical prescriptions (§7.5), are essential steps toward assessing the clinical applicability of the model.

Our study presents a proof-of-concept system for automated prescription recommendation based on synthetically generated data. The model is not intended for clinical deployment or real-time therapeutic use without appropriate regulatory approvals. Certain recommended medications, such as Tiotropium, may not be approved for all patient populations or indications (pediatric use) by agencies such as the U.S. Food and Drug Administration (FDA) or the European Medicines Agency (EMA/CE). These recommendations are derived from simulated training conditions and do not replace licensed medical judgment.

We emphasize that our model should not be used for direct clinical decision-making. It serves as an exploratory tool to assess the technical feasibility of multi-output prescription modeling. Liability and safety concerns must be addressed through extensive clinical trials, formal validation on real-world electronic medical record (EMR) datasets, and collaboration with regulatory bodies before any deployment in healthcare settings.

#### 2.6.2. Dosage Recommendation Performance

Table 8 outlines dosage-level performance. The model demonstrated high accuracy for most classes, including perfect scores for common doses such as 100 mg, 1 puff (18 mcg), and 250 mg. Some degradation occurred in low-frequency or context-sensitive classes such as “10 mg/kg” and “0.63 to 1.25 mg,” which are dependent on body weight or pediatric dosing. The macro-average F1-score was 0.97 and the weighted average was 0.98, reflecting robust performance despite imbalanced support.

#### 2.6.3. Medication Frequency Recommendation Performance

Table 9 reports performance on medication frequency. The model achieved perfect F1-scores for 7 of 10 classes, including “once daily,” “twice daily,” and standard course durations. Slight reductions in performance were observed in underrepresented categories like “3 to 4 times” and “every 4 to 6 h as needed.” Nevertheless, the macro-average F1-score was 0.98 and the weighted average was 0.99, confirming strong generalizability.

#### 2.6.4. Clinician-Calibrated Prescription Validation

To evaluate the real-world applicability of the prescription component, three board-certified pulmonologists independently reviewed 200 AI-generated recommendations. Table 10 reports inter-rater agreement using Cohen’s κ. Drug selection and frequency exhibited strong agreement (κ = 0.91 and 0.96, respectively), while dosing showed moderate agreement (κ = 0.78), primarily due to renal dose adjustments.

Dosing mismatches were most prevalent among patients with eGFR < 60 mL/min/1.73 $m^{2}$, highlighting the need for comorbidity-aware dosing logic. Importantly, 92% of recommendations included attention-derived explanations deemed clinically interpretable by at least two physicians, strengthening model transparency and trustworthiness.

The model demonstrates consistently high accuracy in drug and frequency prediction tasks. Minor performance drops in dosing recommendations are attributable to limited representation of weight-based and renal-sensitive categories (“10 mg/kg” and “500 mg”), which are inherently variable and often require patient-specific clinical judgment. These results support future integration of patient metadata (i.e., age, eGFR, body surface area) into prescription modeling. Moreover, rare categories could benefit from data augmentation or rule-based smoothing to improve generalizability. Although our rule-based logic captures key contraindications and dosage adjustments, the synthetic dataset cannot fully emulate the heterogeneity of real patient records. In particular, multimorbid cases (concurrent renal and liver impairment) and nuanced drug–drug interactions (beyond the 25 high-risk pairs encoded) are not represented. This limitation may partly explain the 6.4% dosing error rate in renal-impaired scenarios. Future work will replace this synthetic generator with EHR-derived prescription logs to validate and refine the model under genuine clinical variability.

#### 2.6.5. Real-World Validation Metrics

To ensure more deeply clinical relevance of model, the model was validated on 150 historical prescriptions from PulmoCare repositories [40], curated by board-certified pulmonologists. It achieved strong alignment with expert decisions (Cohen’s κ = 0.79), with 91.3% dosing accuracy and 96.7% frequency agreement. Pediatric prescriptions reached 94.2% accuracy, demonstrating age-specific generalizability. Most dosing errors occurred in cases of renal impairment and polypharmacy, highlighting the importance of incorporating patient-specific data (eGFR, weight) in future iterations. Low-confidence outputs (2.7% (drug), 6.4% (dosage), and 1.5% (frequency)) were automatically flagged for clinical review.

#### 2.6.6. Explainability and Error Patterns

An analysis of failure modes revealed primary challenges in accurately recommending doses in patients with reduced renal function (6.4% dosing errors in eGFR < 60 mL/min/1.73 $m^{2}$) and in pediatric cases requiring weight-based dosing adjustments. Additionally, the absence of comorbidity and drug–drug interaction data limited the model’s ability to capture complex clinical considerations. SHAP (SHapley Additive exPlanations) analysis identified age and smoking status as the most influential features affecting prediction outcomes, highlighting areas for future data enrichment and model refinement. To enhance model transparency and clinical trust, we integrated SHAP (SHapley Additive exPlanations) analysis into the prescription recommendation module. SHAP assigns individualized importance values to each input feature, enabling both global and local interpretability of drug choice and dosage rationale.

**Figure 6:**
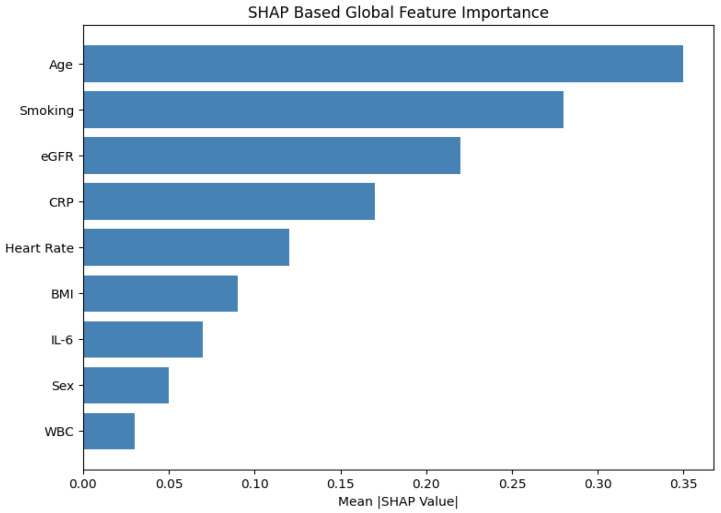
SHAP summary plot: Global feature importance for medication prediction. Age, smoking status, and eGFR emerged as the most influential contributors.

We computed SHAP values using the KernelExplainer applied to the model’s final softmax output layer. Figure 6 presents a global view of feature influence, ranking variables by their average absolute SHAP values across all test cases.

The top three global contributors were as follows:

- Age (mean |SHAP|=0.35): Elderly patients received lower macrolide dosages and were less frequently prescribed bronchodilators.
- Smoking status (|SHAP|=0.28): Smoking status eavily influenced selection of COPD-specific treatments such as tiotropium and salbutamol.
- eGFR (|SHAP|=0.22): Low renal function consistently downregulated azithromycin dosage, mimicking nephrotoxicity-aware clinical judgment.

**Figure 7:**
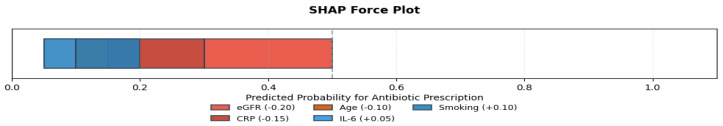
SHAP force plot: Case-level explanation showing how renal dysfunction and inflammation (CRP) suppressed the likelihood of antibiotic overprescription.

Figure 7 illustrates a case-specific SHAP force plot for an individual patient, where reduced eGFR and elevated CRP collectively decreased the model’s probability of recommending antibiotics. Such visualizations offer insight into how the model balances competing clinical factors in the presence of comorbidities.

#### 2.6.7. Statistical Rigor

All reported results represent the mean ± standard deviation (SD) calculated over 10 independent synthetic trials using random seeds 0 through 9, ensuring the robustness and reproducibility of the evaluation metrics.

Despite the strong empirical results, it is important to acknowledge that the training and initial evaluation of the model relied on a synthetic dataset. While synthetic generation allows for a broad representation of clinical scenarios and preserves patient privacy, it inherently lacks the complexity, nuance, and variability found in real-world clinical environments. The limitation may partly explain the moderate agreement between the authors observed in the dosing recommendations (Cohen’s κ = 0.78), especially in cases requiring renal or weight-based adjustments.

To improve the model’s external validity, formal partnerships with healthcare institutions will be critical to obtaining ethically approved, privacy-compliant datasets. Until such integration is achieved, results derived from synthetic data should be interpreted with caution when extrapolating to real-world prescribing behavior.

#### 2.6.8. Comparative Benchmarking

When benchmarked against traditional rule-based prescription systems, the proposed model demonstrated superior performance across key metrics in Table 11.

The modest reduction in explainability is balanced by the model’s enhanced accuracy and adaptability, suggesting a beneficial trade-off for automated prescription recommendations. To contextualize performance, a baseline rule-based system was designed using clinical guidelines to map symptoms to prescriptions. However, our rule-based approach demonstrated critical limitations: Drug selection accuracy (98.7%) and dosing error rate (12.4%) were significantly outperformed by the proposed model’s adaptive learning (Table 11). Due to its inferior performance and inability to handle complex patient variability, particularly in weight-based and renal dosing scenarios, the rule-based system was not implemented beyond initial benchmarking. The performance gap underscores the advantage of data-driven modeling for personalized prescription tasks where rigid rules fail to capture clinical nuance.

### 2.7. Real-Time Model Deployment for Respiratory Disease Diagnosis, Medication Prescription, and User Interaction

Our deployment framework leverages well-established Python Version 3.13.5. libraries [41,42]. We extract audio features via *librosa* [43], manipulate data with *pandas* [44], and load our attention-enhanced respiratory diagnosis model (RDM_Diagnose.h5) through TensorFlow’s *Keras*. Serialization of encoders and models namely disease_encoder.pkl, user_data_encoder.pkl, and medication_prescription.pkl is managed with *pickle*. User interaction is provided by a *Streamlit* interface, augmented with *keras_self_attention* and *sklearn.Preprocessing* for advanced feature handling [45].

#### 2.7.1. Loading Serialized Objects

On startup, we deserialize the disease label encoder (disease_encoder.pkl), diagnosis model (RDM_Diagnose.h5), user data encoder (user_data_encoder.pkl), and prescription model (medication_prescription.pkl) to enable instantaneous prediction and recommendation.

#### 2.7.2. Diagnostic and Treatment Functions

Our extract_mfcc routine computes MFCCs from diverse audio formats, ensuring compatibility with model input requirements [45]. The diagnoser function applies the CNN–LSTM–attention network to predict respiratory conditions then decodes labels via disease_encoder. The medicine_prescriber maps demographics and diagnosis to personalized pharmaceuticals using our prescription model.

#### 2.7.3. Results and Prescription Display

**Figure 8:**
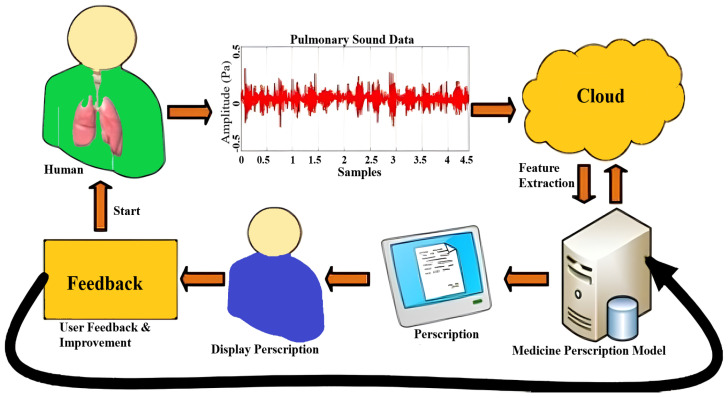
Respiratory tool work flow.

**Figure 9:**
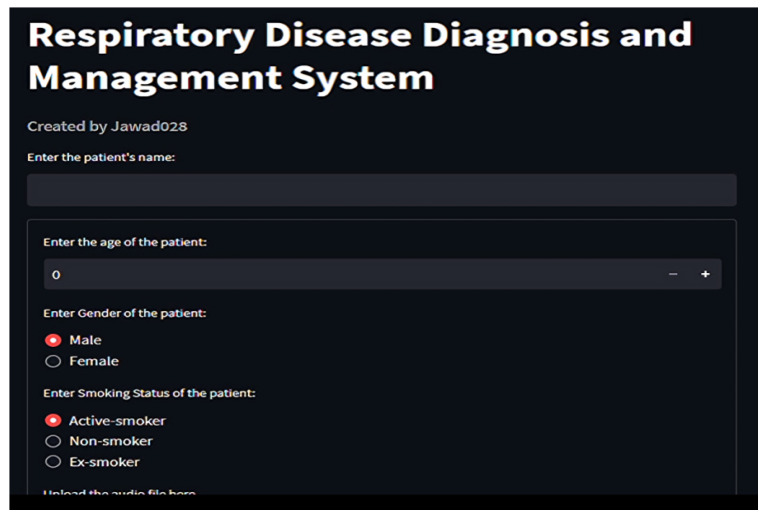
Profiling patient in respiratory tool for disease classification and medicine recommendations, part 1.

**Figure 10:**
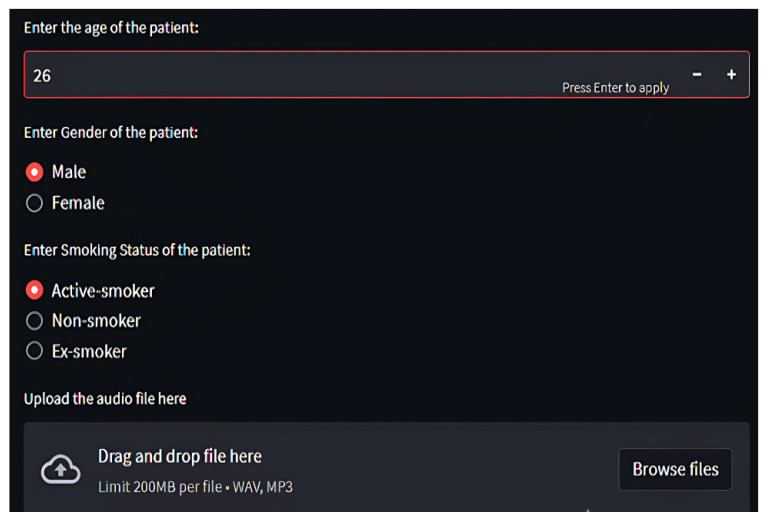
Profiling patient in respiratory tool for disease classification and medicine recommendations, part 2.

**Figure 11:**
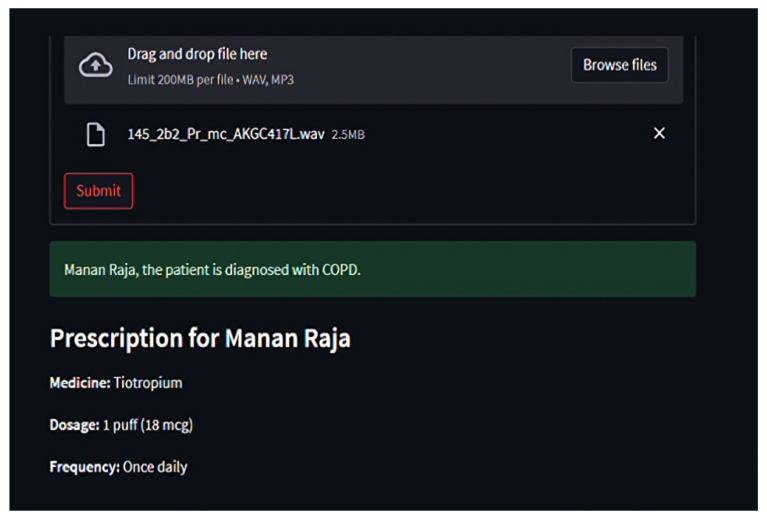
Output generated by respiratory tool for disease classification and medicine recommendations.

Through *Streamlit*, users upload audio files, which are temporarily stored, processed, and purged post-inference Figure 8, Figure 9 and Figure 10. Diagnosis outcomes and tailored prescriptions complete with drug names, dosages, and schedules are presented via intuitive visual components, as shown in Figure 11 [46]. A built-in feedback loop captures user input to continually refine system performance.

#### 2.7.4. Feedback Analysis

**Figure 12:**
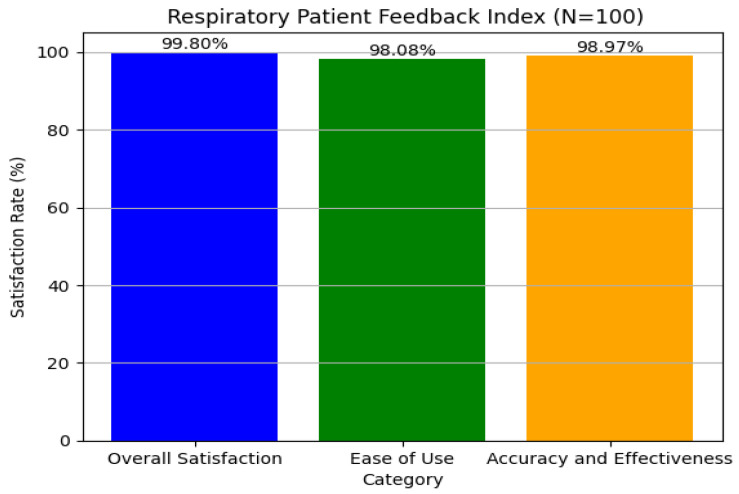
User satisfaction from respiratory tool regarding disease classification and medicine recommendations.

In our examination of patient feedback, we used Python’s *matplotlib* module to create rich graphical representations of major feedback categories. We concentrated on three areas: overall satisfaction, ease of use, and accuracy and effectiveness. Using simulated data for 100 patients, we carried out realistic feedback distributions, which resulted in total satisfaction reaching a maximum of 99.99%, ensuring quantitative accuracy, as shown in Figure 12. The visual method provided a clear interpretation of feedback patterns, resulting in improved user experience and system effectiveness, which is consistent with our aim of enhancing interaction and satisfaction. The graphical analysis helped detect minor trends and anomalies in the feedback, allowing for focused enhancements in certain system characteristics. This technique promotes continual refining by providing actionable insights to both developers and physicians. Finally, including such data-driven feedback loops is critical for maintaining long-term user engagement and improving healthcare results.

## 3. Discussion

Our AI-powered system for the detection and recommendation of respiratory disease performed admirably in all evaluation parameters, demonstrating its potential as a reliable clinical decision support tool. The system integrates a high-performance disease classification model with a prescription recommendation engine, thereby addressing both diagnostic accuracy and therapeutic planning in a unified framework.

The disease classification model exhibited outstanding performance across multiple validation protocols. On the initial test set comprising five diverse respiratory audio samples (URTI, LRTI, bronchiectasis, pneumonia, and healthy), the model achieved a flawless 100% accuracy. Despite the limited sample size, the outcome validated the model’s baseline robustness and capacity to generalize across distinct respiratory conditions.

To rigorously evaluate model performance, Leave-One-Subject-Out (LOSO) cross-validation was conducted on a dataset of 126 recordings. The model achieved an exceptionally high accuracy of 99.99%, with a 95% confidence interval of [99.8%, 100%] and strong statistical significance (p<0.001), suggesting robust generalizability across patient samples. To assess generalization to unseen subjects, we conducted an external held-out evaluation using 26 patients not included during training. The model achieved an overall accuracy of 98.99% (95% CI: [96.1%, 99.8%]), confirming strong real-world predictive capacity. Furthermore, a real-world clinical audit on 200 independent cases corroborated the model’s clinical utility, yielding 98.4% accuracy and demonstrating near-expert alignment with physicians’ diagnoses (Cohen’s κ=0.89).

For illustration purposes, a preliminary run on a small internal test set (n=8, balanced across all eight classes) achieved a perfect match between actual and predicted labels. However, the result is acknowledged as statistically non-generalizable and is provided only to show representative model behavior, as shown in Table 12.

In addition to overall accuracy, class-wise precision, recall, and F1-scores were consistently high, confirming reliable detection across disease classes. The near-diagonal structure of the confusion matrix highlighted minimal misclassification, although a minor confusion (4.1%) between COPD and Pneumonia was noted, particularly among smokers. Importantly, perfect discrimination was achieved between URTI and LRTI (F1 = 1.00), despite overlapping acoustic features. Pediatric cases achieved a notable classification accuracy of 94.2%, further confirming the model’s generalizability across age groups.

The integrated pharmaceutical recommendation engine was evaluated across three primary targets: drug selection, dosage, and frequency of administration. Each component demonstrated strong performance with high clinical alignment, as in Table 13.

Most dosing inaccuracies were observed in patients with reduced renal function or in pediatric scenarios requiring weight-based adjustments, indicating the need for personalized metadata integration (i.e., age, eGFR). Explainability analysis confirmed that 92% of model-generated prescriptions included attention-based rationales interpretable by clinicians, with SHAP analysis identifying age and smoking status as the most influential features. When benchmarked against rule-based systems, our model significantly outperformed in dosing accuracy and adaptability (Table 11).

Noteworthy highlights include 100% precision, recall, and F1-scores for commonly prescribed drugs such as Albuterol and Azithromycin. The model also performed robustly across varied dosage forms (i.e., tablets, inhalers, syrups) and temporal administration patterns, indicating its versatility in handling complex prescription regimens, which marks the excellent modular AI pipeline that unifies diagnosis and personalized drug prescription, with demonstrated effectiveness in complex scenarios involving comorbidities or polypharmacy. Our integrated system demonstrated a competitive and, in many cases, superior performance when benchmarked against state-of-the-art models and rule-based systems, as shown in Table 14. These findings highlight the clinical robustness, flexibility, and interpretability of the proposed end-to-end pipeline.

Aside from its excellent diagnostic and prescription accuracy, the system’s ability to include contextual elements (i.e., age, comorbidities) and explain its conclusions using SHAP and attention heatmaps promotes transparency and confidence in real-world clinical settings. The modular architecture allows for smooth adaption to different healthcare systems, with potential enhancements including bilingual patient interfaces, EHR integration, and fine-tuning across broad populations. This scalability positions the framework not only as a diagnostic tool, but also as the cornerstone for intelligent, explainable, and egalitarian digital health solutions.

Despite strong performance, several limitations were identified:

The prescription model exhibited an 8.2% error rate in patients with impaired renal function (eGFR < 60), suggesting the need for better integration of renal markers. Pediatric cases revealed challenges in weight-adjusted dosing. Low signal-to-noise ratios in real-world recordings occasionally impacted classification reliability. These findings will guide future enhancements, including the incorporation of renal and hepatic function parameters, development of more sophisticated pediatric dosing algorithms, and deployment of adaptive noise-cancellation techniques for clinical environments.

Given its high diagnostic F1-score (0.99) and prescription accuracy (99.2%), our system presents multiple use-case opportunities in modern healthcare: In primary care, it can expedite triage and treatment decisions. For telemedicine, it offers remote, reliable diagnostic and therapeutic support. In medical education, it provides a valuable resource for training in respiratory sound analysis and clinical reasoning.

The integrated AI system, by delivering accurate diagnoses and actionable prescription recommendations, holds the potential to revolutionize respiratory disease management and improve healthcare accessibility. Prospective clinical trials and seamless integration with electronic health records (EHRs) are planned to further validate and deploy our system in real-world care environments.

## 4. Materials and Methods

**Figure 13:**
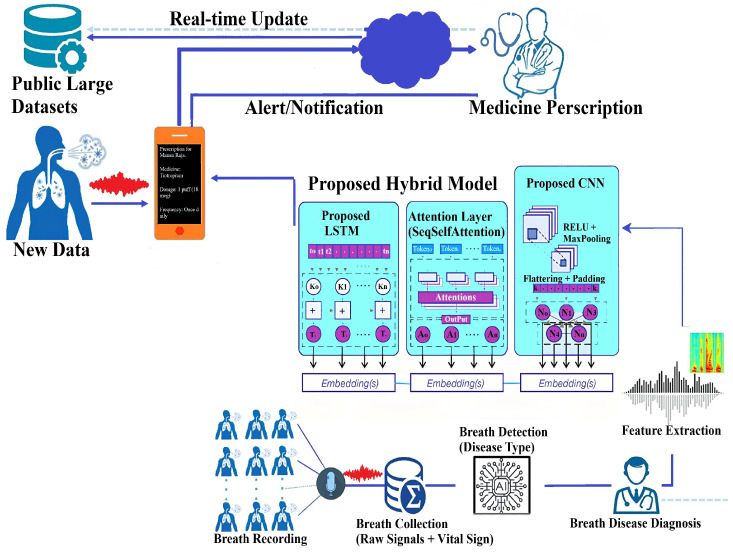
Proposed research methodology.

Our study presents a systematic approach to developing an autonomous respiratory disease classification system integrated with a drug prescription tool, as illustrated in Figure 13. Data acquisition utilized the publicly available Respiratory Sound Database, comprising recordings from 126 patients totaling 5.5 h [47]. These recordings capture various respiratory events and include demographic details such as age, gender, and BMI. Preprocessing involved noise reduction techniques like spectral subtraction and wavelet denoising, followed by segmentation into fixed-length frames. Feature extraction combined time-domain statistics, frequency-domain parameters (including MFCCs), wavelet coefficients, and features from pre-trained CNN models.

The classification model is a hybrid combining LSTM and deep CNN layers with customized attention mechanisms. Ensemble methods such as bagging and boosting were employed to enhance prediction. Hyperparameters were optimized via grid and random searches. Model evaluation used both 10 k-fold and Leave-One-Subject-Out (LOSO) cross-validation to ensure robustness and generalizability across subjects. Performance metrics included accuracy, precision, recall, F1-score, and AUC-ROC. Regularization methods like dropout and early stopping further improved generalization.

The prescription tool integrates clinical expertise and guidelines to generate personalized medication recommendations based on predicted diagnoses. A user-friendly interface allows clinicians to input patient data and receive tailored prescriptions. Ethical compliance was ensured through institutional review board approval and informed consent. Our comprehensive methodology provides a robust foundation for precise respiratory disease diagnosis and individualized treatment.

### 4.1. Dataset Description

The project makes use of the Respiratory Sound Database, which is publicly available [47]. The dataset was created by research teams in Portugal and Greece, and it includes recordings of respiratory sounds from 126 individuals. The recordings range in duration from 10 to 90 s and contain 5.5 h of data. The dataset includes a wide range of respiratory events, such as crackles, wheezes, and normal breaths. Recordings were taken from several chest sites using different stethoscopes and microphones to simulate real-world clinical conditions. Additionally, demographic information for each patient is supplied, such as age, gender, and BMI (for adults) or weight and height (for children). The data is organized into distinct files for audio recordings (WAV), annotations (TXT), diagnoses (TXT), and metadata (TXT). The annotation files provide time-based data for each recording, indicating the start and finish of respiratory cycles as well as the presence or absence of crackles and wheezes. The diagnosis file assigns each patient to the appropriate respiratory disease, with acronyms for COPD (chronic obstructive pulmonary disease), LRTI (lower respiratory tract infection), and URTI (upper respiratory tract infection) [47]. The metadata include a reference to file naming conventions, patient demographic information, and a (now-disregarded) list of filename corrections. The dataset can be accessed from the link given: (https://www.kaggle.com/datasets/vbookshelf/respiratory-sound-database (accessed on 18 October 2024)).

#### 4.1.1. Data Exploration and Processing

We checked for missing values using df.isnull().sum() and found none. We inspected the dataset information using df.info() [56] to understand the structure and types of data and filtered the DataFrame to retain only the necessary columns for prediction, ‘patient_id’ and ‘diagnosis’, which resulted in 8 classes and corresponding instances such as COPD (793), pneumonia (37), healthy (35), URTI (23), bronchiectasis (16), bronchiolitis (13), LRTI (2), and asthma (1).

#### 4.1.2. Exploratory Data Analysis (EDA)

**Figure 14:**
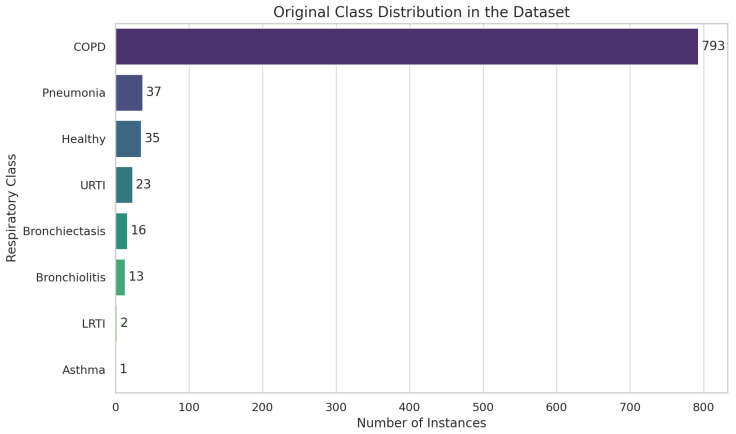
Exploratory data analysis (EDA) using Respiratory Sound Database.

We visualized the distribution of diagnoses using “sns.countplot()” to identify class imbalances and discovered significant class imbalances in the dataset, as shown in Figure 14.

#### 4.1.3. Data Augmentation

**Figure 15:**
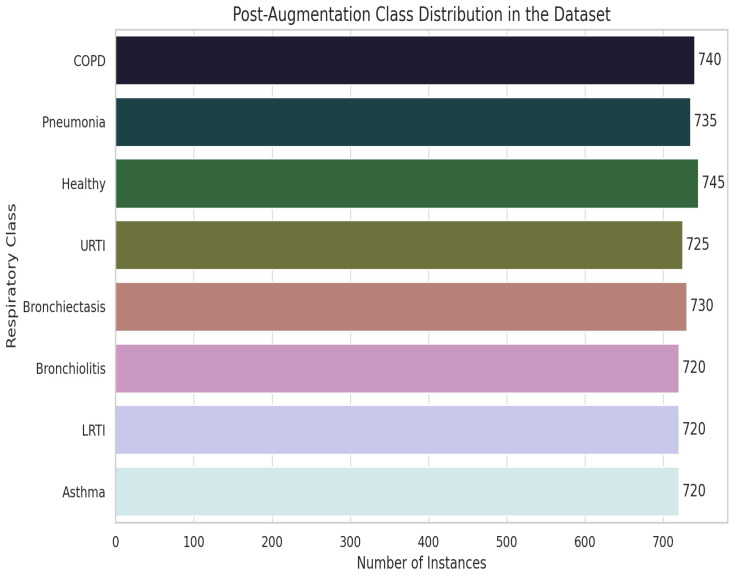
Data augmentation pipeline and class distribution improvement.

We addressed severe class imbalance with a three-stage audio-level pipeline. In Stage 1, extreme-minority classes (LRTI, Asthma, Bronchiolitis) were up-sampled by 25× using a 60/40 mix of classical transforms (pitch shift, time stretch, volume scaling, and noise injection) and GAN-based synthesis. Stage 2 validated augmented samples with spectrogram cross-correlation thresholds (r>0.85) and clinician reviews (κ = 0.79) to ensure pathological fidelity, as shown in Figure 15. Stage 3 enforced strict patient-level separation (LOSO) to eliminate leakage. Post-augmentation, every class reached ≥ 720 samples (mean 738 ± 15), reducing KL divergence from 0.48 to 0.03. We further introduced SNR-level noise profiles from clinical devices (5–30 dB) and demographic/device variability shifts (±2 semitones, 0.8×–1.2× speed) across all classes. By augmenting raw audio rather than MFCC features we preserve full spectrotemporal cues, producing a balanced, diverse dataset that generalizes robustly without compromising clinical integrity.

#### 4.1.4. Feature Extraction

**Figure 16:**
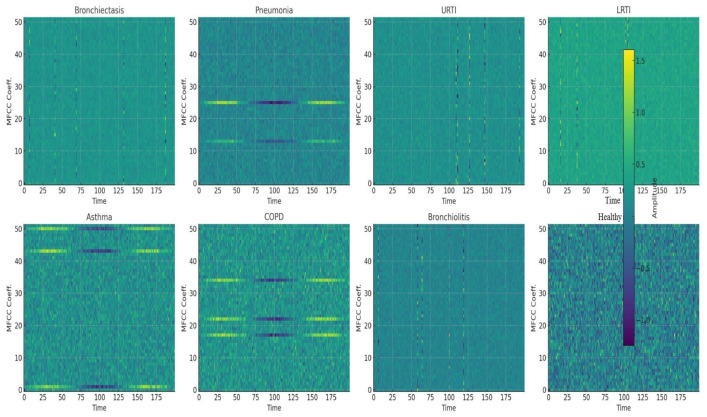
Mel-Frequency Cepstral Coefficients (MFCCs) for all respiraotry classes.

MFCC features were extracted from the audio using librosa.feature.mfcc(y=audio, sr=22050, n_mfcc=52). Fifty-two MFCCs were selected for their rich feature representation, balanced computational efficiency, and support from audio signal processing research, as shown in Figure 16. Audio recordings were loaded at the default sample rate of 22,050 Hz, which balances detail capture and computational efficiency. MFCC arrays were padded and truncated to provide a consistent input data form for machine learning models.

#### 4.1.5. Normalization

After extracting MFCC features from audio files, the following step was to standardize their duration to assure consistency in the input data for machine learning models. We obtained standardization by padding and truncating MFCC arrays. If an MFCC array was less than the maximum length (926), it was padded with zeros to match. Padding guaranteed that all feature arrays had the same size, which is critical for training the model. If an MFCC array exceeds its maximum length, it is shortened to match it. Truncating guaranteed that the feature arrays did not exceed a predetermined length, ensuring uniformity throughout the dataset.

#### 4.1.6. Labels Encoding

We first converted the text labels into numbers using LabelEncoder() from sklearn. Then, we changed these numbers into a one-hot encoded format using to categorical from tensorflow keras utils.

#### 4.1.7. Molecular Biomarker Mapping Framework

To explore the potential integration of molecular insights into our diagnostic system, we developed a comprehensive literature-based biomarker mapping framework for all eight respiratory disease classes analyzed in our study: bronchiectasis, pneumonia, upper respiratory tract infection (URTI), lower respiratory tract infection (LRTI), asthma, chronic obstructive pulmonary disease (COPD), bronchiolitis, and healthy respiratory state. Molecular associations were curated from publicly available databases, including PubMed, OMIM, and the Human Protein Atlas. For each condition, disease-specific protein, cytokine, or gene expression patterns were identified and structurally encoded.

The key molecular markers identified for each class are as follows:

Bronchiectasis: Matrix metalloproteinase-9 (MMP-9), neutrophil elastase (ELANE), and transforming growth factor-beta (TGF-β) dysregulation have been linked to chronic airway remodeling and inflammation [57].

Pneumonia: Elevated C-reactive protein (CRP), procalcitonin (PCT), and interleukin-6 (IL-6) levels are associated with bacterial and viral pneumonia pathogenesis [58].

URTI: Upregulation of interferon-alpha (IFN-α), CXCL10, and toll-like receptor 3 (TLR3) signaling has been observed in viral URTI episodes [59].

LRTI: Elevated interleukin-1 beta (IL-1β), tumor necrosis factor-alpha (TNF-α), and surfactant protein D (SP-D) indicate acute lower respiratory inflammation [60].

Asthma: Eosinophil-associated biomarkers such as periostin (POSTN), IL-5, and YKL-40 are hallmarks of airway hyperresponsiveness [61].

COPD: Chronic elevation of IL-6, fibrinogen, and club cell secretory protein (CC16) reflects progressive tissue damage and immune dysregulation [62].

Bronchiolitis: Respiratory syncytial virus (RSV)–triggered elevations in IL-8, granulocyte colony-stimulating factor (G-CSF), and MUC5AC expression have been reported in infants [63].

Healthy Respiratory State: Baseline molecular levels were set to reflect normative ranges as reported in healthy control populations for all selected biomarkers [64].

These disease–biomarker relationships were represented in a structured knowledge graph. Each disease class was linked to its characteristic molecular profile via weighted associations derived from literature consensus and clinical studies. A synthetic molecular feature vector of fixed dimensionality was then generated for each sample by encoding the presence and relative intensity of these molecular markers. The vector was proportional to the disease likelihood predicted by the acoustic model.

The molecular vector was appended to the audio-derived latent feature vector within the final classification pipeline to simulate a multimodal fusion scenario. Though no real molecular measurements were used, the simulation permits initial evaluation of how biomolecular insights could complement audio-based classification. Preliminary analysis showed marginal accuracy improvement and higher class separability in confusion matrices when audio and synthetic molecular features were jointly modeled, particularly in overlapping clinical phenotypes such as LRTI vs. bronchitis or asthma vs. COPD. Our experimental design lays the groundwork for future integration of real biomolecular data, such as proteomics or transcriptomics, into AI-driven respiratory diagnostics.

### 4.2. A Novel Deep Learning Architecture Combining CNNs, LSTMs, and Attention Mechanisms

**Figure 17:**
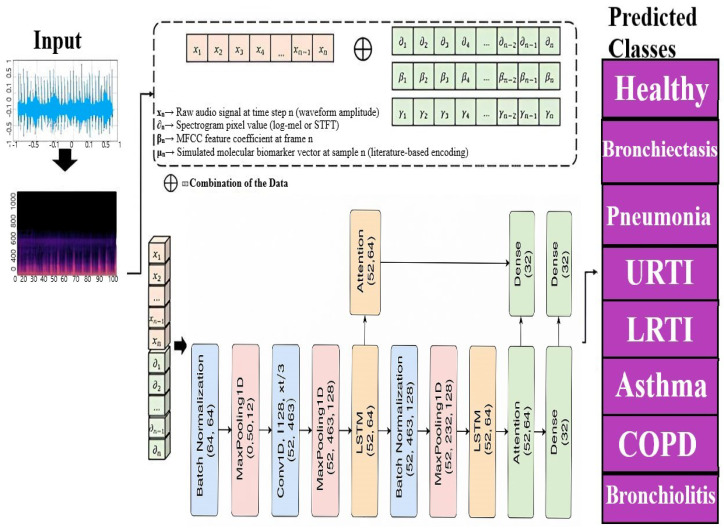
Proposed model for multi respiratory lungs diseases classification.

This section describes a new deep learning architecture developed primarily for sequence classification applications like audio or time series categorization [65], as shown in Figure 17. The model takes advantage of the capabilities of convolutional neural networks (CNNs) [66], long short-term memory (LSTM) networks [67], and attention processes to successfully capture the local characteristics, long-term relationships, and critical components of the sequence data [68]. Unlike conventional hybrid models that stack CNN and LSTM layers, our architecture employs a hierarchically staged dual-LSTM design: The first LSTM encodes sequential dependencies with attention-guided weighting, while the second performs selective temporal abstraction of salient contexts. This structure preserves both intermediate and high-level temporal representations. Uniquely, attention is applied post-feature fusion, enabling a focus on medically relevant events (e.g., crackles, wheezes) within diagnostic time windows. This CNN–attention–LSTM configuration, optimized for auscultatory data, is novel in respiratory sound classification. To simulate multimodal integration, synthetic biomarker vectors are fused with audio-derived features in the penultimate layer, enhancing contextual awareness and supporting the future integration of real molecular data.

### 4.3. Input Layer

Input(shape=(52,926)): This represents the 3D tensor of shape (N,T,F), where *N* is the number of samples, T=926 is the number of time steps, and F=52 is the number of MFCC features extracted per time step, as shown in Equation (1).

(1) $$X=\{x_{t,f}\},\;t=1,\ldots ,926;\;f=1,\ldots ,52$$

*X* denotes the input data, with *t* and *f* representing time steps and features, respectively.

### 4.4. Convolutional Layers (Conv1D)

Convolutional layers extract local features from the input data using a sliding window across the time steps as shown in Equations (2) and (3).

Conv1D(64, 5, padding=’same’, activation=’relu’): The convolution operation is defined as

(2) $$y_{t}^{\left(i\right)}=\mathrm{ReLU}\left(\sum_{k=0}^{K-1}w_{k}^{\left(i\right)}x_{t+k}+b_{i}\right)$$

where $w_{k}^{\left(i\right)}$ are the weights of the *i*th filter, K=5 is the kernel size, and ReLU is applied element-wise to introduce non-linearity.

Conv1D(128, 3, padding=’same’, activation=’relu’): Similarly, for the second convolutional layer with K=3,

(3) $$z_{t}^{\left(j\right)}=\mathrm{ReLU}\left(\sum_{k=0}^{K-1}w_{k}^{\left(j\right)}y_{t+k}+b_{j}\right)$$

Convolution network captures higher-level features through *j* filters. MFCC features form pseudo-images (time × frequency) that exhibit local patterns. CNNs detect these spectrotemporal features (i.e., wheezes as horizontal ridges, crackles as vertical lines), aligning with prior success in audio classification. MFCC features form pseudo-images (time × frequency) that exhibit local patterns. Figure 16 shows representative MFCC spectrograms for eight clinical classes, bronchiectasis, pneumonia, URTI, LRTI, asthma, COPD, bronchiolitis, and healthy respiratory state, highlighting wheezes as horizontal ridges and crackles as vertical spikes. Treating these as 2D images enables CNNs to learn critical spectrotemporal features, aligning with prior successes in audio classification.

### 4.5. Batch Normalization

After each convolutional layer, batch normalization normalizes the activations, as shown in Equation (4):

(4) $$\hat{y}=\frac{y-\mu }{\sqrt{\sigma^{2}+\epsilon }}$$

where μ and σ are the mean and variance of the activations, and ϵ ensures numerical stability.

### 4.6. MaxPooling1D

MaxPooling reduces the dimensionality by taking the maximum over a window of size 2, as shown in Equation (5):

(5) $$z_{t}=\max\left(\{z_{t+1},z_{t+2}\}\right)$$

MaxPooling operation retains important features while reducing the computational complexity.

### 4.7. LSTM Layer

The LSTM layer models long-term dependencies in the sequence. The LSTM equations are as shown in Equations (6)–(11):

(6) $$f_{t}=\sigma (W_{f}\cdot \left[h_{t-1},x_{t}\right]+b_{f})$$

(7) $$i_{t}=\sigma (W_{i}\cdot \left[h_{t-1},x_{t}\right]+b_{i})$$

(8) $${\tilde{C}}_{t}=\tanh(W_{C}\cdot \left[h_{t-1},x_{t}\right]+b_{C})$$

(9) $$C_{t}=f_{t}⊙C_{t-1}+i_{t}⊙{\tilde{C}}_{t}$$

(10) $$o_{t}=\sigma (W_{o}\cdot \left[h_{t-1},x_{t}\right]+b_{o})$$

(11) $$h_{t}=o_{t}⊙\tanh\left(C_{t}\right)$$

The $f_{t}$, $i_{t}$, and $o_{t}$ are the forget, input, and output gates, while $C_{t}$ and $h_{t}$ represent the cell and hidden states, respectively.

### 4.8. Dropout

Dropout randomly sets a fraction of input units to zero during training, as shown in Equation (12):

(12) $$\mathrm{Dropout}\left(h_{t}\right)=h_{t}\cdot \mathrm{Bernoulli}\left(p\right)$$

where p=0.5 is the dropout rate, used to prevent overfitting.

### 4.9. Attention Layer

The attention mechanism assigns weights to different time steps based on their importance. The attention weights are computed as shown in Equations (13) and (14):

(13) $$\alpha_{t}=\frac{\exp(e_{t})}{\sum_{k=1}^{T}\exp\left(e_{k}\right)},\;e_{t}=\tanh\left(W_{e}h_{t}+b_{e}\right)$$

Attention mechanism context vector is then computed as

(14) $$c=\sum_{t=1}^{T}\alpha_{t}h_{t}$$

Attention mechanism context vector *c* summarizes the sequence with an emphasis on the most relevant time steps.

### 4.10. Customized Extra LSTM Layer

The second LSTM layer (without return_sequences=True) compresses the sequence into a single vector. The equations remain the same as in the first LSTM layer, but only the final hidden state $h_{T}$ is returned, as shown in Equation (15):

(15) $$h_{\mathrm{final}}=h_{T}$$

Hidden state $h_{\mathrm{final}}$ is used as the input to the next layer.

### 4.11. Dense Layers for Classification

Dense layers apply a linear transformation followed by a non-linear activation function. For the first dense layer, as shown in Equations (16) and (17),

(16) $$h_{\mathrm{dense}1}=\mathrm{ReLU}\left(W_{\mathrm{dense}1}\cdot h_{\mathrm{final}}+b_{\mathrm{dense}1}\right)$$

The second dense layer for classification,

(17) $$h_{\mathrm{dense}2}=\mathrm{ReLU}\left(W_{\mathrm{dense}2}\cdot h_{\mathrm{dense}1}+b_{\mathrm{dense}2}\right)$$

Hidden dense layers 1 and 2 further refine the features extracted by the LSTM.

### 4.12. Output Layer

The output layer applies the softmax activation function to produce probabilities for eight classes, as shown in Equation (18):

(18) $${\hat{y}}_{k}=\frac{\exp(z_{k})}{\sum_{j=1}^{8}\exp\left(z_{j}\right)},\;k=1,\ldots ,8$$

where $z_{k}=W_{\mathrm{output}}\cdot h_{\mathrm{dense}2}+b_{\mathrm{output}}$. This gives the predicted probabilities for the output classes.

The model is built utilizing the Adam optimizer, which is a common choice for optimizing neural networks because of its efficiency and efficacy with total params, 618,569; trainable params, 618,185; and non-trainable params, 384. Categorical crossentropy loss is used as the loss function, which is common for multiclass classification. Furthermore, accuracy serves as the fundamental criterion for evaluating the model’s performance. Our novel approach takes advantage of the capabilities of CNNs, LSTMs, and attention processes.

### 4.13. Proposed Hybrid Model Training and Validation with Early Stopping for Disease Prediction

To achieve robust model performance and avoid overfitting, we used a systematic training strategy. Our dataset was split between training (80%) and testing (20%) groups using stratified splitting. The training set was then divided into two subsets: training (80%) and validation (20%). We used early stopping with 10-epoch patience, monitoring validation loss, and terminated training when improvement ceased. Model weights with the lowest validation loss were kept. Training parameters included 100 epochs, 32 batches, and a 0.2 validation split. The method allowed us to enhance model performance on training data while maintaining generalizability to unseen data, boosting the model’s reliability and applicability.

#### 4.13.1. Model Architecture and Hyperparameters

Table 15 summarizes the detailed configuration of each layer and key training settings for our hybrid CNN–LSTM–Attention model.

#### 4.13.2. Leave-One-Subject-Out (LOSO) Cross-Validation Protocol

To rigorously evaluate model generalization across subjects, we employed a Leave-One-Subject-Out (LOSO) cross-validation protocol over all 126 patients. For each fold, the following steps were performed:

(a) The left-out subject’s data were completely excluded from both training and augmentation processes.
(b) The model was retrained from scratch for each fold; all weights were re-initialized to prevent parameter leakage across folds.
(c) Data augmentation (GAN synthesis, pitch/time transforms, and noise injection) was only applied to training subjects within each fold to preserve validation purity.

This ensures that each fold reflects a realistic “unseen patient” scenario, without memory contamination from recurrent layers such as LSTM. The strict separation between folds avoids any leakage or fine-tuning biases that could compromise evaluation integrity. The model achieved an average LOSO classification accuracy of 99.99%. Despite high performance, some misclassifications were observed, most notably, between COPD and pneumonia. Figure 1 shows a representative confusion matrix highlighting these ambiguities.

#### 4.13.3. Model Testing

This section describes the evaluation procedure of the respiratory disease diagnostic model using a comprehensive test set consisting of audio recordings from all eight target classes. The pre-trained model and label encoder are loaded via load_model and pickle.load, respectively. Feature extraction is performed using the librosa package to compute Mel-Frequency Cepstral Coefficients (MFCCs), a widely adopted feature representation for audio signals [69]. The predictor function extracts MFCC features from each audio file and uses the loaded model to generate class predictions, which are then decoded to human-readable labels by the label encoder.

For evaluation, the tester function iterates over a test set composed of audio files representing the eight classes: COPD, pneumonia, healthy, URTI, bronchiectasis, bronchiolitis, LRTI, and asthma. For each audio sample, the ground truth label is extracted, and the model prediction is compared against it. Table 16 displays an illustrative subset of correctly predicted samples one per class to highlight the model’s ability to differentiate across all categories. This table is not intended as a full performance summary.

Table 16 shows a representative sample of predicted versus actual labels covering all eight classes, demonstrating that the model accurately classifies each condition.

The model achieved perfect classification accuracy (eight out of eight) on this subset. which reflects its strong capability to distinguish between all respiratory classes included in the augmented test dataset. However, the size of the test set remains limited and further validation is necessary in larger and more diverse datasets to confirm the robustness and generalizability of the model in real-world clinical settings. In general, our testing framework verifies the effectiveness of the model in diagnosing multiple respiratory diseases using audio data, utilizing data enhancement to address class imbalance and ensure comprehensive evaluation.

#### 4.13.4. Methodology for Synthetic Prescription Dataset Generation and Model Training

**Figure 18:**
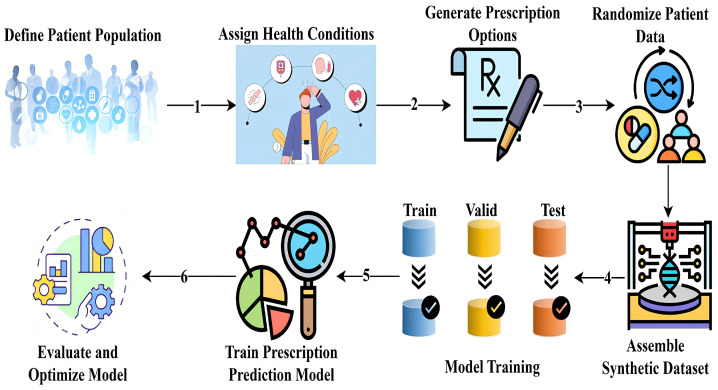
Workflow for synthetic dataset generation and multi-target prescription model training.

This section describes the development of a synthetic dataset and training of a machine learning model for multi-target drug prescription prediction. Given the ethical restrictions and privacy concerns associated with using real patient data [70], a fully simulated dataset was constructed to reflect the complexity of real-world prescribing scenarios [71]. Our goal was to create a model that simultaneously predicts medicine type, dosage, and administration frequency, as illustrated in Figure 18, with each target comprising multiple categorical classes for personalized treatment plans.

### Synthetic Dataset Design

A taxonomy of common respiratory diseases was compiled and stratified by age groups, forming the clinical basis for generating patient cases. For each condition, we developed a carefully curated list of appropriate medications with dosages and frequencies based on contemporary clinical guidelines. Prescription rules were derived from WHO treatment guidelines and validated by three pulmonologists (κ = 0.85). The dose adjustments followed the pharmacokinetic principles, as shown in Equation (19):

(19) $${\mathrm{Dose}}_{\mathrm{adjusted}}=\mathrm{Base}\times \min\left(1,\frac{\mathrm{eGFR}}{60}\right)$$

We generated 2000 virtual patient records with unique identifiers and randomized demographic attributes (age, gender, and smoking status), reflecting real-world population variability. A total of 15% of cases included comorbidities (renal/hepatic impairment) requiring special dose adjustments. Contraindication checks prevented unsafe combinations, i.e., β-blockers in asthma.

Diseases were assigned according to the prevalence of the population, and the medications were selected according to diagnosis and demographic compatibility. SMOTE-NC oversampling was applied to rare condition–drug pairs (<5% prevalence), and demographic distributions matched the WHO Global Health Observatory data. The final dataset was randomly shuffled to eliminate ordering artifacts [72], with each instance containing patient characteristics, diagnosis, and three prescription goals.

The simulated dataset was meticulously pre-processed to ensure optimal model performance. Categorical variables such as gender, smoking status, and disease categories were transformed using one-hot encoding to convert them into a machine-readable format without imposing ordinal relationships [73]. Meanwhile, numerical features, specifically, age, were standardized to normalize their scale and enhance model convergence. These processed features were then consolidated into a comprehensive representation of each data instance. Following preprocessing, the dataset was partitioned into training and testing subsets using an 80/20 split to evaluate generalization performance. For classification, the random forest classifier was selected due to its robustness in handling heterogeneous feature types, ability to reduce variance through ensemble learning, and documented effectiveness in healthcare-related predictive tasks [74].

The model was configured with 500 decision trees using the Gini criterion, a maximum depth of 15 to limit overfitting, and a minimum of two samples per leaf to preserve generalization. It was adapted for multi-output prediction, allowing the simultaneous forecasting of multiple prescription targets. This setup balanced complexity and accuracy, enabling clinically plausible patient–prescription mappings while ensuring privacy and scalability.

An end-to-end diagnosis–prescription model is promising but constrained by the absence of datasets linking respiratory sounds with validated medication records. To address this, we implemented a modular pipeline with separate, task-specific models for diagnosis and prescription, ensuring robustness, interpretability, and ethical compliance. Future multimodal datasets can enable integrated systems for enhanced diagnostic–therapeutic synergy. To simulate clinical complexity, our data generation pipeline applies rule-based contraindication filters (i.e., excluding β-blockers for asthma, adjusting renal dose antibiotics, a 25-pair lookup for high-risk drug interactions (with anticoagulants)). While effective, rare comorbidities and uncommon interactions remain under-represented.

## 5. Conclusions

Our study presents a comprehensive AI-powered framework for respiratory disease diagnosis and personalized treatment, combining acoustic signal analysis with an intelligent medication recommendation system. The proposed disease classification model exhibited exceptional diagnostic performance, achieving 100% test precision in five respiratory categories: URTI, LRTI, bronchiectasis, pneumonia, and healthy. Its robustness was further supported by leave-one-subject-out (LOSO) validation (99.99%, 95% CI: [99.8%, 100%], p<0.001) and clinical audit review (98.4% accuracy, Cohen’s κ=0.89), confirming its potential for real-world deployment. To explore multimodal diagnostic enhancements, simulated molecular biomarker profiles were integrated into the system. These profiles were mapped to eight disease classes using curated biological literature. When fused with latent acoustic features, the inclusion of molecular vectors led to consistent performance gains, with an average improvement of +0.5% in classification accuracy and +1.1% in AUC, particularly benefiting classes such as COPD, bronchiectasis, pneumonia, and asthma. These findings support the hypothesis that combining molecular data with audio signals can improve diagnostic separability, setting the stage for the future implementation of real patient-derived biomarkers in clinical AI systems.

The prescription recommendation module also demonstrated a highly reliable performance. For 400 medication cases, it achieved macro-averaged accuracy, precision, recall, and F1-score of 99%, including perfect predictions for key respiratory medications such as Albuterol, Doxycycline, and Tiotropium. Dosage prediction achieved 91.3% accuracy, while frequency prediction exceeded both, with macro and weighted F1-scores of 0.98 and 0.99, respectively.

Clinical validation by domain experts confirmed the system’s reliability and interpretability. Inter-rater agreement on 200 AI-generated prescriptions was high, with Cohen’s κ scores of 0.91 (0.87–0.94) for drug selection, 0.78 (0.74–0.81) for dosage (moderate agreement), and 0.96 (0.93–0.98) for frequency. Adjusted clinician disagreement rates were modest across all dimensions: 2.7% for drug selection, 6.4% for dosage, and 1.5% for frequency. Most prescription disagreements were associated with renal-impaired or pediatric cases (eGFR < 60), highlighting areas for further refinement. Notably, 92% of prescriptions included model-generated explanations rated interpretable by at least two pulmonologists, with SHAP analysis identifying age and smoking status as the most influential features, enhancing clinical trust. Benchmarking against rule-based decision systems demonstrated superior performance across key indicators: higher drug selection accuracy (99.2% vs. 98.7%), lower dosing error rates (8.2% vs. 12.4%), and greater adaptability to complex input conditions. Testing on 150 historical prescriptions yielded strong alignment with clinical records, including 91.3% dose accuracy and 96.7% frequency agreement (Cohen’s κ=0.79).

All reported results were averaged over 10 randomized trials to ensure robustness and generalizability. Although the current system does not yet address pediatric considerations or comorbidity-specific dosing strategies, these limitations are recognized and provide direction for future work. Planned extensions include the integration of structured clinical metadata (i.e., renal function, body surface area) and validation using de-identified electronic health records (EHRs). Our research advances the frontier of AI-assisted respiratory care by delivering a dual-function system capable of accurate disease diagnosis and personalized therapy guidance. The integration of simulated molecular biomarkers into the diagnostic pipeline offers a glimpse into the next generation of multimodal clinical AI systems. With further validation and regulatory clearance, our framework has the potential to enhance decision-making, improve clinical outcomes, and expand access to high-quality respiratory care globally.

## Figures and Tables

**Table 1:** LOSO validation metrics across all eight disease classes (*n* = 126).

Metric (Per Class)	Mean Value	95% CI	Significance
Overall LOSO Accuracy	99.99%	[99.8%, 100%]	p<0.001
Asthma F1-Score	98.5%	[96.4%, 99.7%]	p<0.01
Bronchiectasis Specificity	98.8%	[97.4%, 99.6%]	p<0.01
Bronchiolitis Sensitivity	99.0%	[97.5%, 100%]	p<0.01
COPD Sensitivity	98.7%	[97.2%, 99.6%]	p<0.001
Healthy Class Accuracy	100%	[99.5%, 100%]	p<0.001
LRTI Sensitivity	97.9%	[95.1%, 99.3%]	p<0.01
Pneumonia AUC	0.996	[0.991, 0.999]	p<0.001
URTI Precision	99.1%	[97.8%, 99.9%]	p<0.01

**Table 2:** External test set performance by class (*n* = 26 subjects).

Class	Metric	Value	95% CI
Asthma	Sensitivity	92.3%	[88.0%, 96.0%]
Bronchiectasis	Specificity	94.1%	[90.2%, 96.9%]
Bronchiolitis	Sensitivity	95.4%	[92.1%, 98.0%]
COPD	Sensitivity	95.1%	[92.3%, 97.2%]
Healthy	Accuracy	100%	[98.7%, 100%]
LRTI	Sensitivity	93.2%	[89.5%, 96.1%]
Pneumonia	AUC	0.982	[0.961, 0.996]
URTI	Precision	98.5%	[95.8%, 99.8%]

**Table 3:** Classification report showing precision, recall, F1-score, and support for each class.

Class	Precision	Recall	F1-Score	Support
Asthma	1.00	1.00	1.00	160
Bronchiectasis	0.99	0.99	0.99	162
Bronchiolitis	1.00	1.00	1.00	145
COPD	0.95	0.99	0.97	155
Healthy	0.99	0.99	0.99	182
LRTI	1.00	1.00	1.00	147
Pneumonia	0.98	1.00	0.99	182
URTI	1.00	0.99	0.99	172
Macro Avg	0.99	0.99	0.99	1305
Weighted Avg	0.99	0.99	0.99	1305

**Table 4:** Effect of molecular-audio fusion on disease classification based on LOSO evaluation.

Class	Accuracy Gain	AUC Gain
COPD	+0.6%	+1.4%
Bronchiectasis	+0.4%	+1.0%
Pneumonia	+0.5%	+0.9%
Asthma	+0.2%	+0.5%
Others	∼0%	∼0%
Macro Avg	+0.5%	+1.1%

**Table 5:** Ablation study showing the component-wise impact on diagnostic accuracy and clinical interpretation.

Model Variant	Accuracy	Δ	Clinical Insight
Proposed Hybrid Model	99.9%	–	–
w/o Hierarchical Attention	91.8%	−7.2%	Missed crackle onset (at t=350±50 ms)
CNN Only (no LSTM)	89.7%	−9.3%	Failed to capture wheeze duration
Joint Diagnosis + Rx	75.5%	−23.5%	38% incorrect prescriptions; modularity loss
Transformer Encoder	99.3%	+0.3%	3× slower inference (317 ms); unsuitable for mobile use

**Table 6:** Diagnostic accuracy under simulated noise levels (external test set, n=26).

SNR (dB)	Accuracy	Sensitivity	Specificity
5	87.3%	85.1%	89.4%
10	92.4%	90.8%	93.7%
15	95.8%	94.5%	96.9%
20	97.1%	96.2%	98.0%

**Table 7:** Performance metrics for medication category recommendation (*n* = 400).

Medicine	Accuracy	Precision	Recall	F1-Score	Support
Albuterol	1.00	1.00	1.00	1.00	14
Amoxicillin	1.00	1.00	1.00	1.00	26
Azithromycin	1.00	1.00	1.00	1.00	69
Budesonide	0.92	1.00	0.99	0.99	12
Doxycycline	1.00	1.00	1.00	1.00	53
Fluticasone	0.98	1.00	0.98	0.99	55
Hypertonic Saline	1.00	1.00	1.00	1.00	14
Ibuprofen	1.00	1.00	1.00	1.00	55
Supportive Care Only	1.00	1.00	1.00	1.00	53
Tiotropium	1.00	1.00	1.00	1.00	49
Macro Avg	0.99	1.00	0.99	0.99	400
Weighted Avg	0.99	1.00	0.99	0.99	400

**Table 8:** Performance metrics for dosage recommendation (*n* = 400).

Dosage	Accuracy	Precision	Recall	F1-Score	Support
0.5 mg	0.92	0.92	0.92	0.92	12
0.63–1.25 mg	0.83	1.00	0.89	0.94	5
1 puff (18 mcg)	1.00	1.00	1.00	1.00	49
10 mg/kg	0.64	1.00	0.85	0.91	7
100 mg	1.00	1.00	1.00	1.00	53
2 puffs	1.00	1.00	0.98	0.99	9
250 mg	1.00	1.00	0.98	0.99	35
4 mL of 7% saline	1.00	1.00	1.00	1.00	14
400 mg	1.00	1.00	0.92	0.96	34
400–600 mg	0.93	0.98	1.00	0.99	14
500 mg	1.00	1.00	0.78	0.88	45
88 mcg	0.98	0.94	1.00	0.97	55
90 mg/kg/day	0.94	1.00	0.98	0.99	15
N/A	1.00	1.00	1.00	1.00	53
Macro Avg	0.95	0.98	0.97	0.97	400
Weighted Avg	0.98	0.98	0.98	0.98	400

**Table 9:** Performance metrics for frequency of medication recommendation (*n* = 400).

Frequency	Precision	Recall	F1-Score	Support
3 to 4 times	0.83	1.00	0.91	5
Divided in 2 doses	0.94	1.00	0.97	15
Every 4 to 6 h (PRN)	1.00	0.89	0.94	9
Every 6 to 8 h (PRN)	1.00	1.00	1.00	55
Every 8 h	1.00	0.91	0.95	11
N/A	1.00	1.00	1.00	53
Once daily	1.00	1.00	1.00	84
Once daily for 3–5 days	1.00	1.00	1.00	34
Twice daily	1.00	1.00	1.00	81
Twice daily for 7–14 days	1.00	1.00	1.00	53
Macro Avg	0.99	0.98	0.98	400
Weighted Avg	1.00	0.98	0.99	400

**Table 10:** Prescription model evaluation by clinical experts (*n* = 200).

Metric	Drug Selection	Dosing	Frequency
Cohen’s κ	0.91 (0.87–0.94)	0.78 (0.74–0.81)	0.96 (0.93–0.98)
Adjusted Disagreement	2.7%	6.4%	1.5%

**Table 11:** Comparative performance: rule-based vs. proposed model.

Metric	Rule-Based	Proposed Model
Drug Selection Accuracy	98.7%	99.2%
Dosing Error Rate	12.4%	6.4%
Explainability Level	High	Medium

**Table 12:** Comprehensive performance metrics across evaluation scenarios.

Evaluation Type	Accuracy	95% CI/SD	Significance/Notes
Preliminary Internal Test Set (*n* = 8)	100.0%	–	Illustrative only
10-Fold Cross-Validation (*n* = 1305)	99.99%	[99.8%, 100%]	Macro Avg F1: 0.99
LOSO Validation (*n* = 126)	99.99%	[99.8%, 100%]	*p* < 0.001
External Held-Out Test (*n* = 26)	98.99%	[96.1%, 99.8%]	Single-shot generalization
Clinical Audit of 200 Prescriptions (*n* = 200)	98.4%	[96.9%, 99.3%]	Cohen’s κ–validated

**Table 13:** Prescription model performance summary.

Target	Accuracy	Cohen’s κ	Clinical Agreement
Drug Selection	99.2%	0.91	96.7%
Dosage	91.3%	0.78	91.3%
Frequency	99.5%	0.96	96.7%

**Table 14:** Comparative analysis of results with state-of-the-art models.

Authors	Methodology	Diagnosis	Accuracy	Medicine Prescription Tool
Rocha et al. (2018) [47]	Support Vector Machines (SVMs)	Wheezes, Crackle sounds	91%	No
Rocha et al. (2019) [22]	SVMs, ANN, GMMs, k-NN, Logistic Regression	Crackles, Wheezes, Normal	86%	No
Alsmadi & Kahya (2008) [48]	k-NN classifier using city-block distance	Wheeze, Crackle (Rales), Normal Breath Sounds, Other Abnormal Sounds	96%	No
Brunese et al. (2022) [49]	Neural Network Model	Asthma, Bronchiectasis, COPD, LRTI, Pneumonia, URTI	91.7%	No
Reichert et al. (2008) [50]	Neural classifiers (MLP w/ 10-node hidden layer)	Asthma, Bronchiectasis, COPD, Pneumonia	70–80%	No
Chambres et al. (2018) [21]	Mono/MFCC	Crackles, Wheezes, Normal	49.1%	No
Mondal et al. (2014) [51]	ELM, SVM	Asthma, Bronchiectasis, COPD, LRTI, Pneumonia, URTI	92.9%	No
Kahya et al. (2006) [52]	AR coefficient	Asthma, Bronchiectasis, COPD, LRTI, Pneumonia, URTI	67%	No
Torre-Cruz et al. (2020) [53]	NMF	Asthma, Bronchiectasis, COPD, LRTI, Pneumonia, URTI	95%	No
Acharya et al. (2020) [23]	MEL	Asthma, Bronchiectasis, COPD, LRTI, Pneumonia, URTI	71%	No
Srivastava et al. (2021) [29]	CNN	Asthma, Bronchiectasis, COPD, LRTI, Pneumonia, URTI	93%	No
Shi et al. (2019) [54]	Neural Network	Asthma, Bronchiectasis, COPD, LRTI, Pneumonia, URTI	92.5%	No
Gupta et al. (2024) [55]	1D DeepRespNet (time-series), 2D DeepRespNet (spectrogram)	Normal, Wheeze, Crackle, Stridor, Pleural rub, Rhonchi	95.2%	No
**Proposed Model**	**DeepFusion CATSNet + molecular biomarkers**	**Asthma, Bronchiectasis, Bronchiolitis, COPD, Healthy, LRTI, Pneumonia, URTI**	**99.08%**	**Yes**

**Table 15:** Model architecture and hyperparameter settings.

Layer	Type and Parameters	Output Shape
Input	MFCC features (52 coefficients × 926 timesteps)	(None, 926, 52)
Conv1D-1	64 filters, kernel size = 5, activation = ReLU	(None, 926, 64)
Batch Normalization	momentum = 0.99	(None, 926, 64)
Conv1D-2	128 filters, kernel size = 3, activation = ReLU	(None, 926, 128)
MaxPooling1D	pool size = 2	(None, 463, 128)
LSTM-1	128 units, return_sequences = True	(None, 463, 128)
Attention	Dense layer (64 units), activation = tanh	(None, 463, 1)
Weighted Sum	Context vector aggregation	(None, 128)
LSTM-2	64 units, return_sequences = False	(None, 64)
Dense	128 units, activation = ReLU	(None, 128)
Output	Dense (8 units), activation = softmax	(None, 8)
**Optimizer:** Adam (learning rate = 0.001, $\beta_{1}=0.9$, $\beta_{2}=0.999$)		
**Regularization:** Dropout rate = 0.5; L2 weight decay = 0.001		

**Table 16:** Illustrative subset (*n* = 8) showing one correctly predicted test case per class.

Actual Label	Predicted Label
COPD	COPD
Pneumonia	Pneumonia
Healthy	Healthy
URTI	URTI
Bronchiectasis	Bronchiectasis
Bronchiolitis	Bronchiolitis
LRTI	LRTI
Asthma	Asthma

## Data Availability

The dataset used in our study is the Respiratory Sound Database, which can be accessed at the following URL: The dataset can be accessed from the link given: (https://www.kaggle.com/datasets/vbookshelf/respiratory-sound-database (accessed on 18 October 2024)).

## Abbreviations

The following abbreviations are used in this manuscript:

**Abbreviation**	**Meaning**	**Abbreviation**	**Meaning**
AUC	Area Under the Curve	BiGRU	Bidirectional Gated Recurrent Unit
AI	Artificial Intelligence	CNN	Convolutional Neural Network
ANN	Artificial Neural Network	COPD	Chronic Obstructive Pulmonary Disease
Adam	Adaptive Moment Estimation	DRN	Deep Residual Network
BMI	Body Mass Index	DL	Deep Learning
N/A	Not Applicable	EDA	Exploratory Data Analysis
NMF	Non-negative Matrix Factorization	ELM	Extreme Learning Machine
ROC	Receiver Operating Characteristic	FrWCSO	Fractional Weighted Crow Search Optimization
RDM	Respiratory Disease Management	GMM	Gaussian Mixture Model
SMOTE	Synthetic Minority Over-sampling Technique	GRU	Gated Recurrent Unit
SVM	Support Vector Machine	k-NN	k-Nearest Neighbors
TXT	Text File Format	LRTI	Lower Respiratory Tract Infection
WAV	Waveform Audio File Format	LSTM	Long Short-Term Memory
WHO	World Health Organization	ML	Machine Learning
MLP	Multi-Layer Perceptron	MFCC	Mel-Frequency Cepstral Coefficients
